# Genome-Wide Identification and Stress-Responsive Expression Analysis of the PR-1 Gene Family in *Paulownia fortunei*

**DOI:** 10.3390/plants15203160

**Published:** 2026-10-15

**Authors:** Shaokang Guo, Bingxu Chen, Xibing Cao, Na Guo, Guoqiang Fan, Hui Zhao

**Affiliations:** 1 College of Forestry, Henan Agricultural University, Zhengzhou 450002, China; guoshaokang17@163.com (S.G.); 15689099437@163.com (B.C.); cxb-1106@163.com (X.C.); guona@henau.edu.cn (N.G.); 2 Institute of Paulownia, Henan Agricultural University, Zhengzhou 450002, China

**Keywords:** *Paulownia fortunei*, *PfPR-1*, phytoplasma infection, abiotic stress, ERF1, transcriptional regulation

## Abstract

Pathogenesis-related protein 1 (*PR-1*) genes are involved in plant defense responses and abiotic stress responses. In this study, a total of 14 *PfPR-1* family members were identified from the genome of *Paulownia fortunei*, and their physicochemical properties, phylogenetic relationships, conserved motifs, promoter *cis*-acting elements, chromosomal localization, and syntenic relationships were systematically analyzed. The results showed that *PfPR-1* family members exhibited considerable variation in protein physicochemical characteristics and structural composition, suggesting evolutionary and functional divergence among different members. Promoter analysis revealed the presence of numerous hormone-responsive, stress-responsive, and light-responsive *cis*-acting elements, indicating that *PfPR-1* genes may be involved in multiple biological processes and signaling pathways. Transcriptome sequencing analysis demonstrated that most *PfPR-1* genes significantly responded to Paulownia witches’ broom (PaWB) disease, salt stress, and drought stress. Under phytoplasma infection, five *PfPR-1* genes were markedly upregulated (*PR-1-1*, *PR-1-3, PR-1-4, PR-1-8,* and *PR-1-14*), whereas four *PfPR-1* genes (*PfPR-1-6*, *PfPR-1-7*, *PfPR-1-12*, and *PfPR-1-13*) were downregulated, suggesting their differential responses to phytoplasma infection. Under salt- and drought-stress conditions, different *PfPR-1* genes exhibited diverse expression patterns, indicating their potential functional specialization in stress-responsive regulation. Subsequent qRT-PCR analysis further confirmed the reliability of the transcriptome data. Notably, the expression of *PR-1-3* was significantly upregulated under both biotic and abiotic stresses. Yeast one-hybrid and dual-luciferase reporter assays demonstrated that an ERF transcription factor (TF), PfERF1, could significantly activate the transcription of *PfPR-1-3*, suggesting that PfERF1 may participate in stress-responsive regulation in *Paulownia fortunei* through modulating *PfPR-1* expression. Collectively, these findings provide a theoretical basis for further investigation of the biological functions and molecular regulatory mechanisms of the *PfPR-1* gene family during stress responses in *Paulownia fortunei*.

## 1. Introduction

Plants are continuously exposed to various biotic stresses, including fungal, bacterial, and viral pathogens, as well as abiotic stresses such as extreme temperatures, drought, and salinity. During the long process of evolution, plants have gradually developed complex and precise defense and stress-response systems. Pathogenesis-related proteins (PRs) are important components of plant defense mechanisms against pathogen invasion and abiotic stresses. The PR protein family members are highly diverse in both structure and function and have been classified into 17 subfamilies (i.e., PR-1~PR-17) according to their structural and functional characteristics. Among them, members of the PR-1, PR-4, PR-5, and PR-10 subfamilies have been demonstrated to play critical roles in disease resistance, abiotic stress tolerance, and the biosynthesis of secondary metabolites, such as harringtonine in *Cephalotaxus hainanensis* [1,2,3,4].

The PR-1 subfamily was first identified in tobacco that was infected with Tobacco mosaic virus in the late 1960s, and it is one of the earliest and most extensively studied PR protein families. PR-1 proteins play critical roles in plant immunity and are widely regarded as molecular markers of systemic acquired resistance (SAR) [1]. Recently, increasing evidence indicates that PR-1 proteins not only function as direct antimicrobial proteins but also as immune signaling regulators. For example, the C-terminal CAP-derived peptide (CAPE) released from *Arabidopsis thaliana* PR-1 can activate immune responses and enhance disease resistance [5]. Similarly, the CAPE1 peptide derived from tomato (*Solanum lycopersicum*) PR-1 inhibits the growth of *Ralstonia solanacearum*, thereby improving resistance against bacterial wilt [6,7]. In rice (*Oryza sativa*), overexpression of *OsPR-1a* and *OsPR-1b* can significantly enhance plant resistance to rice blast and bacterial diseases [8].

Recent studies further demonstrated that PR-1 proteins also play important roles in disease resistance and abiotic stress adaptation in woody plants. In poplar (*Populus deltoides*), the *PdePR-1-10* gene is strongly induced following rust pathogen infection, and its overexpression can significantly enhance rust resistance in transgenic plants under the regulation of WRKY TFs, indicating an important role of *PR-1* genes in salicylic acid (SA)-dependent immune pathways in woody species [9]. In grape (*Vitis vinifera*), *VvPR-1* genes are demonstrated to be closely associated with resistance to powdery mildew and downy mildew [10]. In eucalyptus (*Eucalyptus grandis*), EgPR-1 and other defense-related proteins rapidly respond to fungal infection and participate in pathogen recognition, reactive oxygen species (ROS) accumulation, and systemic acquired resistance [11]. These findings suggest that PR-1 proteins perform functions in both direct antimicrobial defense and immune signal regulation in woody plants.

In addition to biotic stress responses, PR-1 proteins also have been reported to be related to plant tolerance to abiotic stresses such as drought and salinity. In apple (*Malus domestica*), MdPR-1 family members respond to both *Botryosphaeria dothidea* infection and salt stress, and their expression is coordinately regulated by SA and jasmonic acid (JA) signaling pathways [12]. In tomato, *PR-1* family genes exhibit differential expression patterns under drought conditions, indicating their involvement in drought tolerance [13]. In *Arabidopsis*, salt and drought treatments significantly induce the expression of *AtPR-1* through ABA-dependent pathways, thereby enhancing drought-stress tolerance [14]. Moreover, JA-mediated activation of *PR-1* has also been reported to improve the salt tolerance of plants [15]. These studies collectively suggest that PR-1 proteins may function as important regulatory nodes connecting biotic and abiotic stress signaling pathways [16]. In woody plants, abiotic stress responses exhibit more complex and long-term regulatory characteristics, and the associated transcriptional regulatory networks still require further in-depth elucidation [17].

*Paulownia fortunei*, a perennial deciduous tree belonging to the family Paulowniaceae, is native to China and widely distributed in the Yangtze River Basin and southern China [18,19]. Due to its rapid growth, high-quality timber, strong adaptability, and remarkable ecological value, it has been extensively used in timber production, agroforestry systems, and ecological restoration [20,21]. However, Paulownia witches’ broom (PaWB), caused by phytoplasma infection, severely threatens the development of the Paulownia industry. PaWB-infected plants exhibit leaf yellowing, witches’ broom symptoms, dwarfism, and growth stagnation, resulting in severe economic losses [22,23]. In addition to biotic stresses, salinity and drought are also major environmental factors limiting Paulownia growth and productivity. High salinity and water deficiency disrupt cellular osmotic balance and ROS metabolism, thereby inhibiting plant growth and reducing forests’ ecological benefits [24,25].

According to our previous transcriptome sequencing results, several *PfPR-1* homologs were differentially expressed under phytoplasma infection, salt stress, and drought stress, suggesting that the *PfPR-1* family may participate in the coordinated response of *Paulownia fortunei* to both biotic and abiotic stresses. Although PR-1 genes have been extensively characterized in several herbaceous plants and woody species, the genome-wide identification, evolutionary characteristics, and coordinated responses of PR-1 family members to multiple stresses in *Paulownia fortunei* remain largely unexplored. In particular, the potential functional differentiation of *PfPR-1* members in response to phytoplasma infection, salt stress, and drought stress, as well as their upstream transcriptional regulatory mechanisms, has not yet been systematically investigated. Therefore, this study aimed to (i) identify and characterize the PR-1 gene family in *P. fortunei* through genome-wide analysis, including analyses of physicochemical properties, phylogenetic relationships, gene structures, conserved motifs, *cis*-acting elements, chromosomal localization, and syntenic relationships; (ii) determine the expression patterns of *PfPR-1* genes under phytoplasma infection, salt stress, and drought stress using transcriptome and qRT-PCR analyses; and (iii) identify and experimentally validate transcriptional regulators of a representative stress-responsive *PfPR-1* gene. Among these genes, *PfPR-1-3*, which exhibited pronounced responses to both biotic and abiotic stresses, was selected for further investigation. Yeast one-hybrid and dual-luciferase reporter assays demonstrated that the transcription factor PfERF1 directly binds to the *PfPR-1-3* promoter and activates its transcription, providing preliminary evidence for an ERF-mediated regulatory mechanism of *PfPR-1-3*. Collectively, this study provides a systematic characterization of the *PR-1* gene family in *Paulownia fortunei* and extends our understanding of PR-1-mediated stress responses in woody plants by linking family-wide stress-responsive expression with upstream transcriptional regulation. The identified PfERF1–*PfPR-1-3* regulatory module provides a potential molecular framework for understanding the integration of biotic and abiotic stress responses and offers candidate genetic resources for improving disease resistance and abiotic stress tolerance in *Paulownia fortunei*.

## 2. Results

### 2.1. Physicochemical Characteristics of PfPR-1 Family Members in Paulownia fortunei

A total of 14 *PfPR-1* genes were identified from the *Paulownia fortunei* genome and were named *PfPR-1*-*1* to *PfPR-1*-*14* according to their chromosomal locations. The physicochemical properties of the *PfPR-1* proteins are summarized in Table 1. The PfPR-1 proteins range from 147 amino acids (*PfPR-1-14*) to 226 amino acids (*PfPR-1-5*) in length, with predicted molecular weights of 17.02–25.85 kDa, indicating a relatively conserved size distribution within the family. The theoretical isoelectric points (pI) vary from 5.24 (*PfPR-1-11*) to 9.37 (*PfPR-1-12*), suggesting diversity in charge properties among PfPR-1 members. The instability index values range from 26.17 (*PfPR-1-4*) to 50.46 (*PfPR-1-11*); notably, the majority of PfPR-1 proteins exhibit instability index values below 40, suggesting that more than half of PfPR-1 members are predicted to be relatively stable under in vitro conditions, while the other PfPR-1 proteins (*PfPR-1-2*, *PfPR-1-8*, *PfPR-1-9*, *PfPR-1-11*, *PfPR-1-12* and *PfPR-1-13*) with high instability index values (greater than 40) may be unstable in vitro. Notably, all PfPR-1 proteins exhibit negative GRAVY (grand average of hydropathicity) values (−0.459 to −0.215), demonstrating that they are predominantly hydrophilic, which is consistent with the typical characteristics of soluble PfPR-1 proteins. Subcellular localization prediction revealed that PfPR-1 proteins are mainly localized in the chloroplast and extracellular region, while a few members are localized in the mitochondrion or cytoplasm. The diverse predicted localization patterns among PfPR-1 members may be associated with differences in their amino acid sequences, particularly N-terminal targeting sequences or other sequence features that determine intracellular trafficking. These differences suggest that individual PfPR-1 members may have distinct cellular distributions. However, as these results were obtained based on computational prediction, further experimental validation is required to confirm the actual subcellular localization of each PfPR-1 protein.

### 2.2. Phylogenetic Analysis of PfPR-1 Family Members

**Figure 1:**
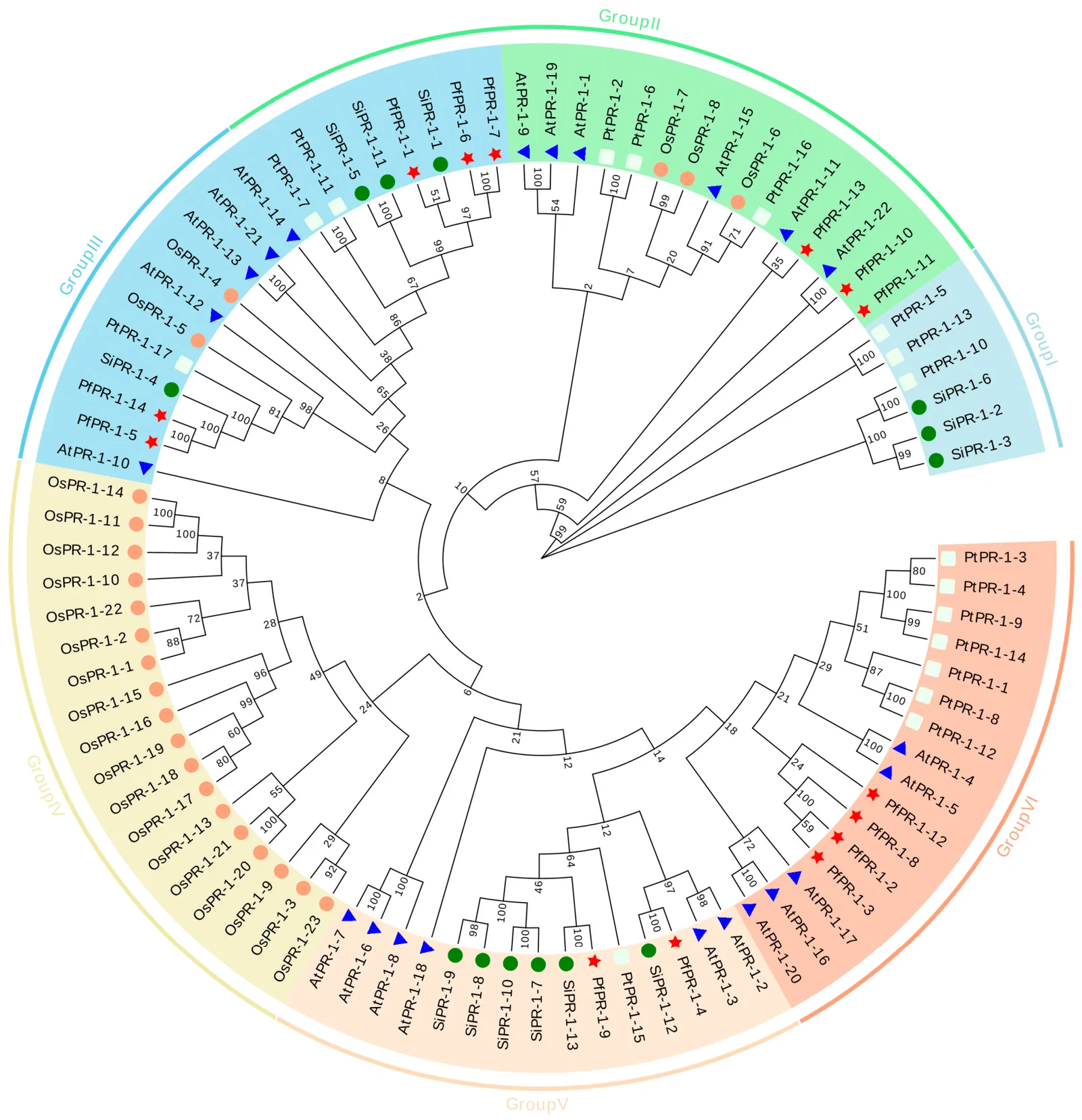
Phylogenetic analysis of PR-1 proteins from five representative plant species. The phylogenetic tree was constructed based on PR-1 protein sequences from *Paulownia fortunei*, *Arabidopsis thaliana*, *Populus trichocarpa*, *Sesamum indicum*, and *Oryza sativa* using the neighbor-joining method with 1000 bootstrap replicates. Bootstrap values (%) are indicated at the corresponding branch nodes to evaluate the reliability of the phylogenetic relationships. The PR-1 proteins were classified into six major clades (Groups I–VI) according to the topology of the phylogenetic tree. Major clades were further supported by maximum likelihood analysis. Different colors and symbols represent PR-1 proteins from different plant species.

To investigate the evolutionary relationships of PR-1 proteins among different plant species, a phylogenetic tree was constructed using PR-1 protein sequences from *Paulownia fortunei*, *Arabidopsis thaliana*, *Populus trichocarpa*, *Sesamum indicum*, and *Oryza sativa*. Based on the topology of the phylogenetic tree, PR-1 proteins were classified into six major clades (Groups I–VI) (Figure 1).

Group I represented a relatively small clade mainly containing PR-1 proteins from *Populus trichocarpa* and *Sesamum indicum*. The clustering of *PtPR-1-5*, *PtPR-1-13*, and *PtPR-1-10* with *SiPR-1* members indicated that this lineage may represent a conserved PR-1 subgroup among woody and herbaceous dicot species. Group II consisted of PR-1 proteins from multiple plant species, including *Arabidopsis*, *Populus*, *Oryza*, and *Paulownia*. Several *PfPR-1* proteins, including *PfPR-1-10*, *PfPR-1-11*, and *PfPR-1-13*, were distributed in this group, suggesting that these members share evolutionary relationships with PR-1 proteins from other angiosperm species. Group III was the largest clade and contained PR-1 proteins from all five examined species. Multiple *PfPR-1* proteins, including *PfPR-1-1*, *PfPR-1-5*, *PfPR-1-6*, *PfPR-1-7*, and *PfPR-1-14*, were clustered in this group together with homologous proteins from *Arabidopsis*, *Populus*, *Sesamum*, and *Oryza*. The broad species distribution of this group suggested that these PR-1 members represent highly conserved evolutionary lineages. Group IV was composed exclusively of PR-1 proteins from *Oryza sativa*. The presence of multiple OsPR-1 members in this clade suggested lineage-specific diversification of PR-1 genes within monocot species. Group V contained PR-1 proteins from *Arabidopsis*, *Paulownia*, *Populus*, and *Sesamum*. The distribution of *PfPR-1-4* and *PfPR-1-9* together with homologous proteins from other dicot species indicated evolutionary conservation of this PR-1 lineage among dicot plants. Group VI contained PR-1 proteins from all examined dicot species, including multiple *PfPR-1* members (*PfPR-1-2*, *PfPR-1-3*, *PfPR-1-8*, and *PfPR-1-12*). Several closely related homologous pairs were identified, such as *PfPR-1-3* and *PtPR-1-3*, suggesting that these PR-1 genes may have originated from conserved ancestral genes before species divergence.

Overall, the phylogenetic analysis demonstrated that the PR-1 gene family contains both conserved evolutionary lineages and lineage-specific diversification patterns. The distribution of *PfPR-1* proteins across different clades indicates that PR-1 members in *P. fortunei* have retained evolutionary relationships with homologous proteins from diverse plant species, while also exhibiting potential diversification during plant evolution.

### 2.3. Structure and Conserved Domain Analysis of the PfPR-1 Members in Paulownia fortunei

**Figure 2:**
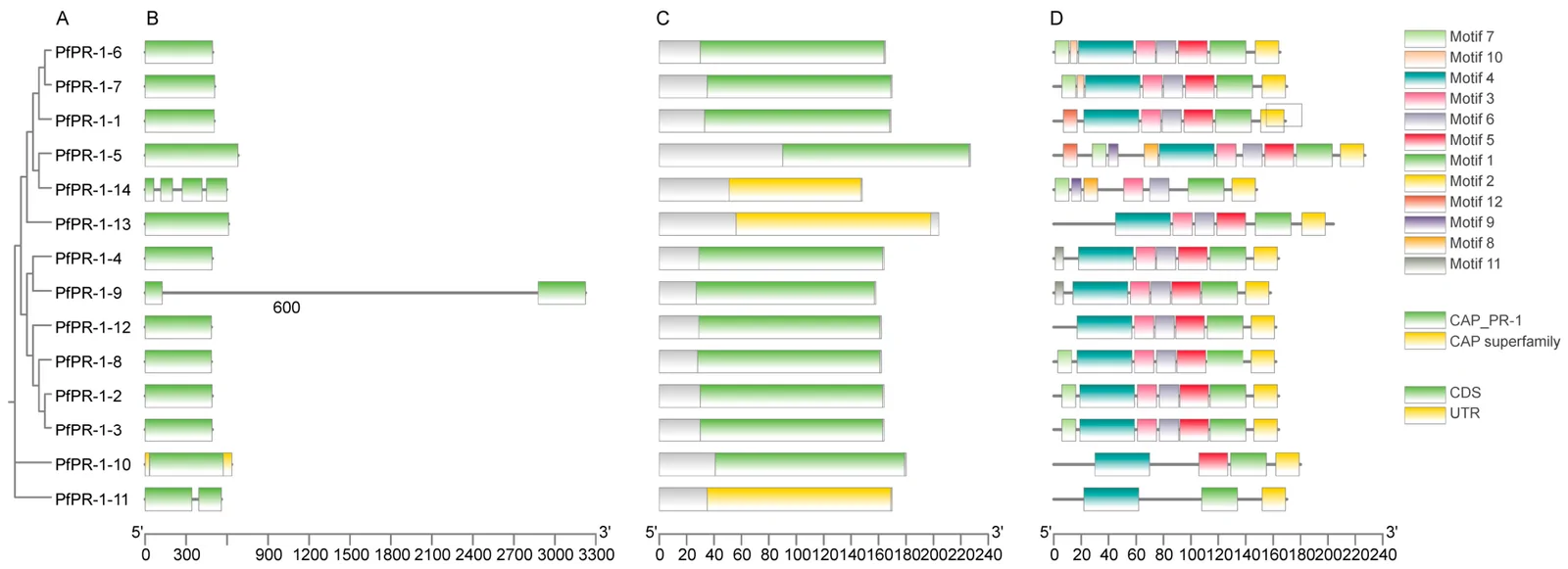
Evolutionary relationships (**A**), gene structures (**B**), conserved domain (**C**) and motifs (**D**) of the *PfPR-1* members in *Paulownia fortunei*.

A genomic structural analysis of the *PfPR-1* gene family clearly classified its members into three categories (Figure 2B). The majority of *PfPR-1* genes (e.g., *PfPR-1-1* to *PfPR-1-8*) possess a typical intronless organization, with the coding region composed of a single continuous exon. In contrast, *PfPR-1-9* and *PfPR-1-11* contain two exons, whereas *PfPR-1-14* harbors four exons, representing the most structurally complex member of the family. Notably, *PfPR-1-10* constitutes a unique case, as it is the only gene identified with partial intronic sequences, suggesting the possibility of incompletely annotated splice variants.

Using the Pfam tool, we predicted the conserved domains of the PfPR-1 protein family (Figure 2C). The result revealed that all members contain a core CAP (Cysteine-rich secretory proteins, Antigen 5 and PR-1) domain. The majority members possess the characteristic CAP-PR-1 domain, demonstrating high sequence conservation among PfPR-1 proteins. However, specific amino acid variations at key positions within this domain were observed in two particular members, *PfPR-1-5* and *PfPR-1-10*. Furthermore, a distinct subset of members (PfPR-1-11, PfPR-1-13, and PfPR-1-14) harbor domains classified under the broader CAP superfamily rather than the typical CAP-PR-1 type, suggesting potential evolutionary or functional divergence in this subgroup. Although typical PR-1 proteins in other species often maintain a highly conserved CAP-PR-1 structure, the PfPR-1 members analyzed here exhibit notable structural variation. For instance, while most members within closely related phylogenetic clades retain the conserved CAP-PR-1 domain, some other members containing the CAP superfamily domain form a distinct cluster. This result reflects a degree of structural plasticity and potential functional specialization during the evolution of the PfPR-1 family.

According to the above results, variation in exon number is not randomly distributed in *PfPR-1* genes but closely associated with protein domain classification: the multi-exon genes (*PfPR-1-11* and *PfPR-1-14*) precisely correspond to members characterized as containing a CAP superfamily domain, whereas most single-exon genes encode proteins with the typical CAP-PR-1 domain. This strong consistency between domain type and gene structure indicates coordinated evolution.

Conserved motif analysis of the PfPR-1 family revealed that all members harbor Motif 1 and Motif 2, which constitute the core structural framework underlying canonical function of PfPR-1 proteins (Figure 2D). The co-occurrence of Motif 3 and Motif 6 defines a predominant subgroup comprising *PfPR-1-1* to *PfPR-1-9* and *PfPR-1-12* to *PfPR-1-14*. In contrast, Motif 4 and Motif 5 exhibit lineage-specific loss patterns: Motif 4 is absent only in *PfPR-1-14*, whereas Motif 5 is specifically absent in *PfPR-1-11* and *PfPR-1-14*. Notably, these motif-deficient members precisely correspond to the proteins that were previously identified as containing a CAP superfamily domain (Figure 2C) and a relatively complex multi-exon gene structure (Figure 2B), indicating a tight association among motif composition, domain architecture, and gene structural organization. Such coordinated variation suggests that the PfPR-1 family may have undergone structural diversification that could contribute to functional differentiation. Overall, these findings suggest that the PfPR-1 family has undergone evolutionary diversification characterized by conservation of essential functional elements alongside flexible modification of peripheral structural components.

### 2.4. Cis-Acting Elements Analysis of the PfPR-1 Gene Promoters

**Figure 3:**
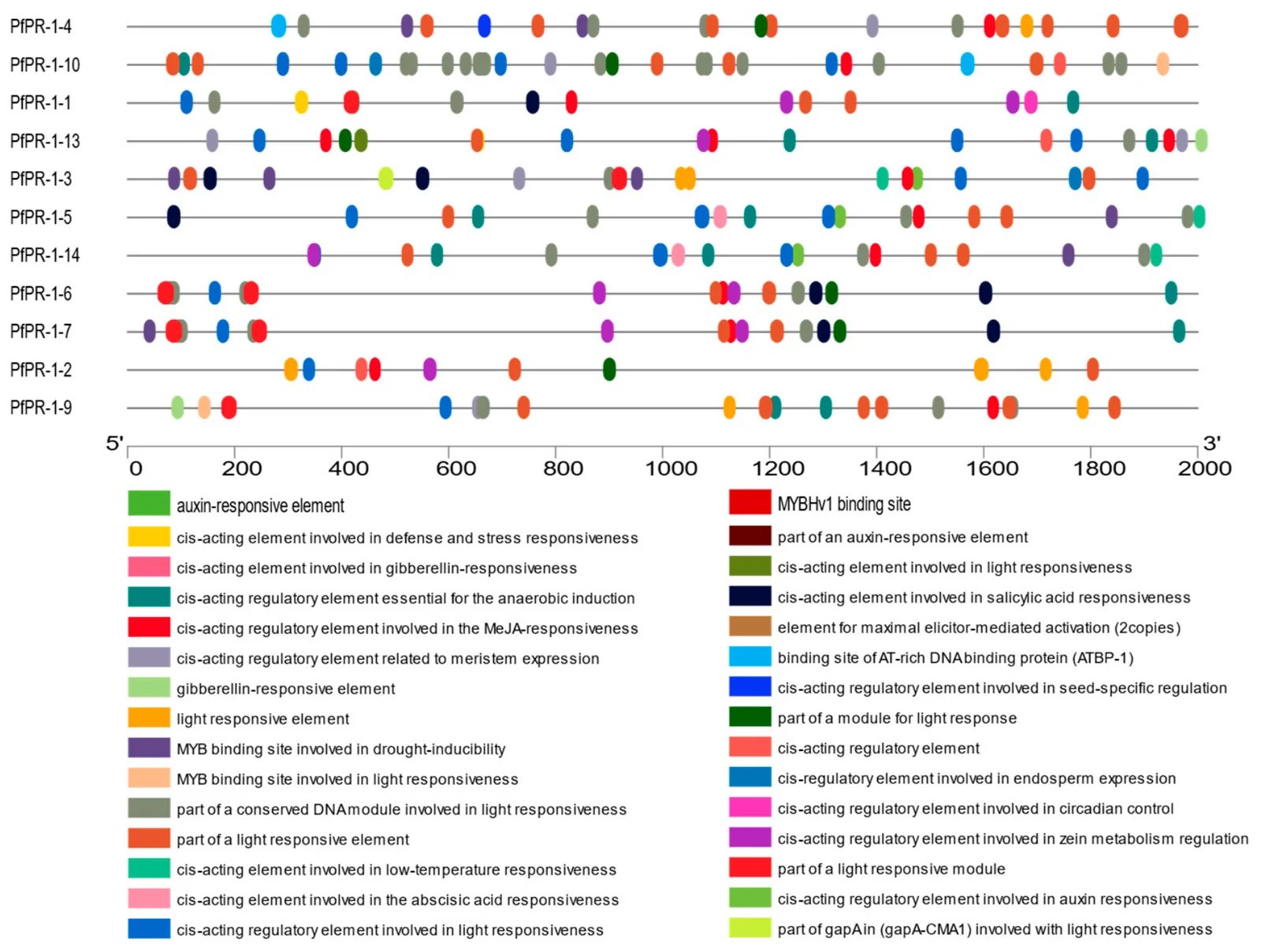
Analysis of *cis*-acting elements in the promoter region of *PfPR-1* genes in *Paulownia fortunei.*

The promoter region plays a crucial role in gene expression regulation by responding to environmental and developmental signals. In this study, we analyzed the *cis*-acting elements in the *PfPR-1* gene promoters, and identified 40 distinct *cis*-acting elements (Figure 3). According to the *Paulownia fortunei* genome, the upstream 2000 bp sequences of *PfPR-1* genes were extracted as the promoter regions. The promoter sequence analysis revealed a complex regulatory landscape associated with multiple signaling pathways. Numerous hormone-responsive elements were identified, including auxin-responsive elements, gibberellin-responsive elements, abscisic acid-responsive elements, methyl jasmonate (MeJA)-responsive elements, and salicylic acid-responsive elements, suggesting that *PfPR-1* genes may participate in extensive hormone-mediated regulatory networks. In addition, several stress-related *cis*-acting elements were detected, such as defense- and stress-responsive elements, anaerobic induction elements, low-temperature-responsive elements, and MYB binding sites involved in drought inducibility, indicating a potential role of *PfPR-1* genes in plant responses to diverse environmental stresses. A large number of light-responsive elements were also present in the *PfPR-1* promoter regions, including canonical light-responsive elements, conserved DNA modules of light-responsive regulatory units, MYB binding sites associated with light signaling, and gapA-related modules involved in light regulation. This enrichment suggests that the transcription of *PfPR-1* genes may be strongly influenced by light signaling pathways. Furthermore, several development-related regulatory elements were identified, including meristem expression elements, seed-specific regulatory elements, endosperm expression elements, and circadian rhythm regulatory elements. Collectively, these findings indicate that the expression of *PfPR-1* genes is probably coordinately regulated by hormonal signals, environmental stresses, light signaling, and developmental cues, providing a molecular basis for their diverse roles in plant growth, development, and stress responses.

### 2.5. Chromosome Localization and Homology Analysis of PfPR-1 Gene Family Members

**Figure 4:**
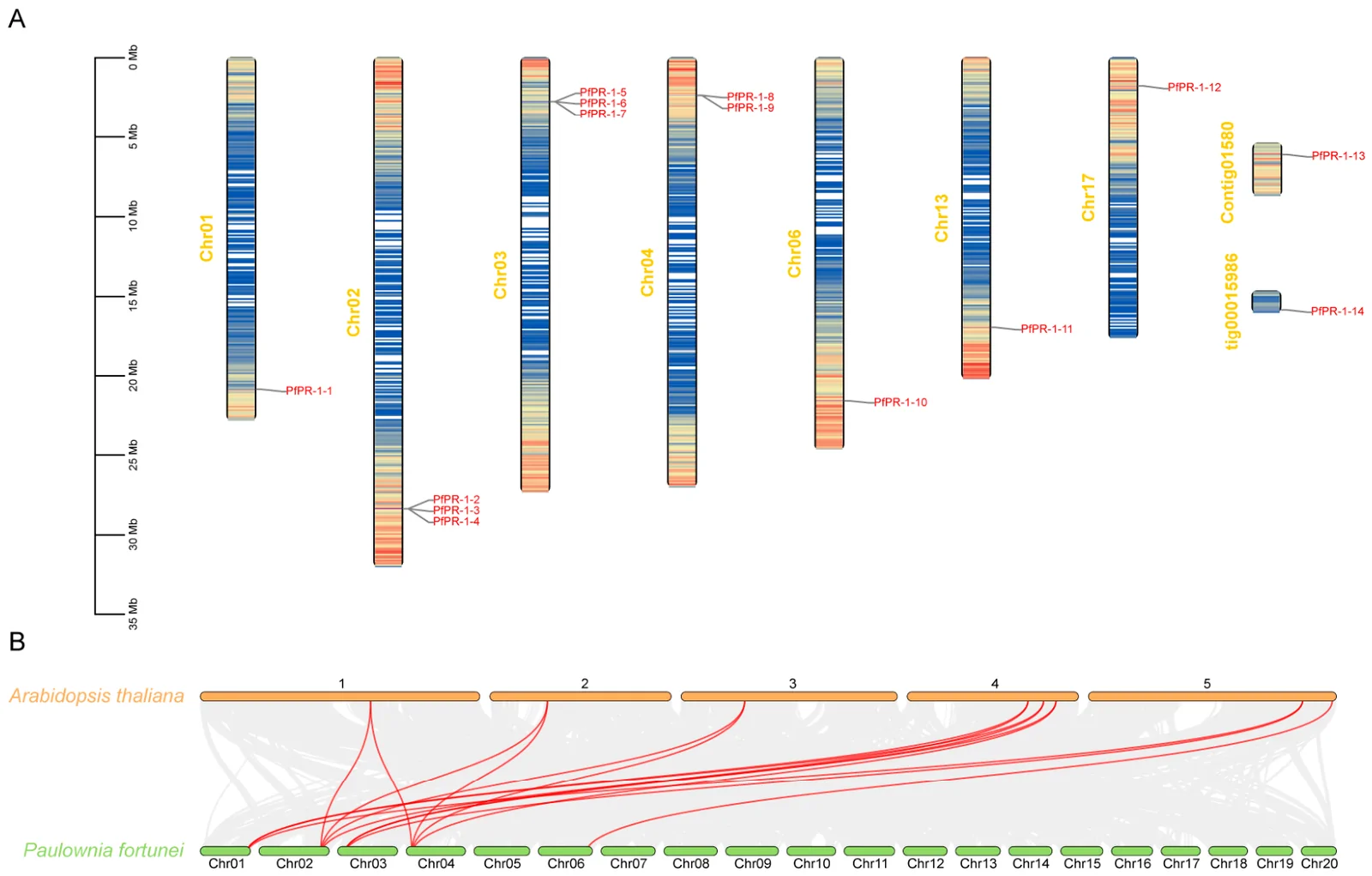
Chromosome distribution and collinearity analysis of *PfPR-1* genes. (**A**): Distribution of *PfPR-1* genes in chromosomes. (**B**): Collinearity analysis of *PR-1* genes between *Paulownia fortunei* and *Arabidopsis thaliana*.

The chromosomal localization analysis of the *PfPR-1* genes showed that 85.71% (12/14) of *PfPR-1* genes were distributed across seven chromosomes, while two genes (*PfPR-1-13* and *PfPR-1-14*) were not anchored to any chromosome. Chromosomes 2 and 3 each harbored three *PfPR-1* genes and Chromosome 4 contained two *PfPR-1* genes, whereas the remaining four chromosomes (Chr01, Chr06, Chr13, and Chr17) each carried only one *PfPR-1* gene, indicating an uneven distribution pattern of the *PfPR-1* gene family in the genome (Figure 4A). In addition, interspecies synteny analysis revealed that *Paulownia fortunei* and *Arabidopsis thaliana* shared 13 syntenic gene pairs, highlighting a relatively close evolutionary relationship between the two species (Figure 4B). This finding suggests that they may share a common ancestral origin.

### 2.6. Expression Analysis of PfPR-1 Genes Under Biotic and Abiotic Stress

**Figure 5:**
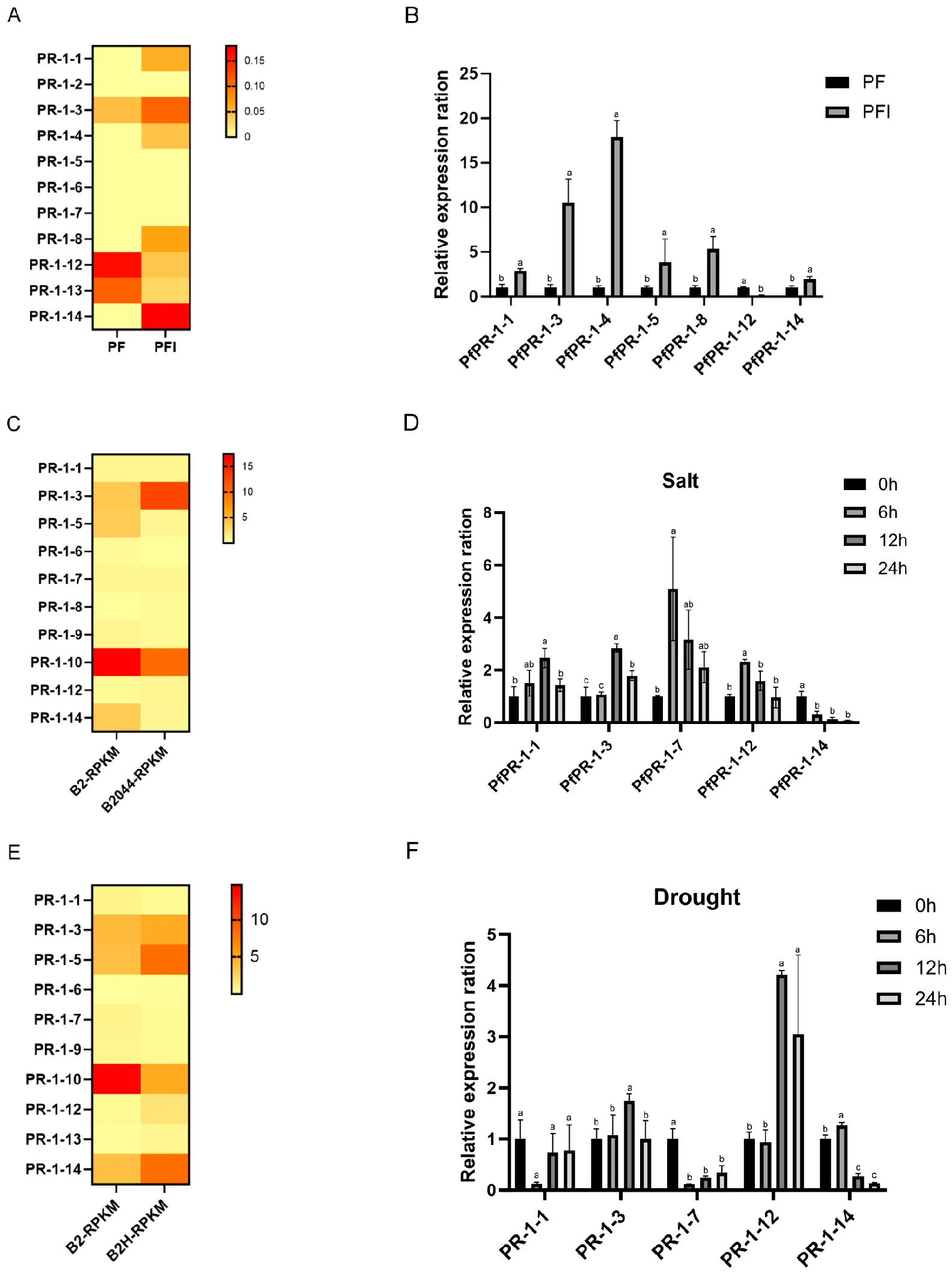
Differential expression profiles of *PfPR-1* genes in *Paulownia fortunei* under biotic and abiotic stresses. (**A**,**C**,**E**) Transcriptome-based expression profiles of *PfPR-1* genes under witches’ broom disease, salt, and drought stresses, respectively. Differentially expressed genes were identified based on the criteria of |log2 (Fold Change)| ≥ 1 and false discovery rate (FDR) < 0.05. PF, healthy *Paulownia fortunei* seedlings; PFI, *Paulownia fortunei* seedlings infected with witches’ broom disease; B2-RPKM, Reads Per Kilobase of transcript per Million mapped reads values for healthy *Paulownia fortunei* seedlings; B2044-RPKM, Reads Per Kilobase of transcript per Million mapped reads values for *Paulownia fortunei* seedlings under salt stress; B2H-RPKM, Reads Per Kilobase of transcript per Million mapped reads values for *Paulownia fortunei* seedlings under drought stress. (**B**,**D**,**F**) qRT-PCR validation of *PfPR-1* gene expression patterns under witches’ broom disease, salt, and drought stresses, respectively. The expression levels were calculated using the 2^−ΔΔCt^ method. Data are presented as mean ± SD from three biological replicates (*n* = 3). Different lowercase letters (a, ab, b, c) indicate significant differences among treatments according to one-way ANOVA followed by LSD and Tukey’s HSD multiple comparisons test (*p* < 0.05). Means sharing at least one common letter are not significantly different, whereas means with no common letters are significantly different.

To explore the transcriptional responses of the *PfPR-1* genes in *Paulownia fortunei* under different stress conditions, the expression patterns of *PfPR-1* genes were extracted and visualized based on previously generated and analyzed RNA-seq datasets from our laboratory [26]. These datasets included transcriptomic data obtained under three treatment conditions, including phytoplasma infection, salt stress, and drought stress. Differentially expressed genes were identified in the original transcriptome analyses using the criteria of |log_2_ (Fold Change)| ≥ 1 and false discovery rate (FDR) < 0.05, and gene expression levels were quantified using FPKM (Fragments Per Kilobase of transcript per Million mapped reads) values. In the present study, the expression profiles of *PfPR-1* genes were extracted from these datasets and visualized by TBtoolsv2.483 after log_2_ (FPKM + 1) transformation and hierarchical clustering (Figure 5A). The results showed that the relative expression levels of *PfPR-1-2* and *PfPR-1-5* showed no significant changes under phytoplasma infection. The expression of *PfPR-1-1*, *PfPR-1-3*, *PfPR-1-4*, *PfPR-1-8*, and *PfPR-1-14* was upregulated, while the expression of *PfPR-1-6*, *PfPR-1-7*, *PfPR-1-12*, and *PfPR-1-13* was downregulated under phytoplasma infection, indicating their potential involvement in the response to PaWB. In the qRT-PCR analysis, representative *PfPR-1* genes showing relatively pronounced expression changes and trends consistent with the transcriptome data were selected for presentation, including *PfPR-1-1*, *PfPR-1-3*, *PfPR-1-4*, *PfPR-1-5*, *PfPR-1-8*, *PfPR-1-12*, and *PfPR-1-14* (Figure 5B). The qRT-PCR results generally supported the expression patterns observed in the transcriptome analysis, highlighting the complex expression responses of *PfPR-1* genes to phytoplasma infection in *Paulownia fortunei*.

The expression patterns of the *PfPR-1* gene family under salt and drought stresses also revealed distinct differences among different genes (Figure 5C,E). Under salt stress, *PfPR-1-1*, *PfPR-1-3*, *PfPR-1-7*, *PfPR-1-8*, and *PfPR-1-12* were upregulated, whereas *PfPR-1-5*, *PfPR-1-6*, *PfPR-1-9*, *PfPR-1-10*, and *PfPR-1-14* were downregulated (Figure 5C). Under drought stress, *PfPR-1-3*, *PfPR-1-5*, *PfPR-1-6*, *PfPR-1-12*, *PfPR-1-13*, and *PfPR-1-14* were upregulated, while *PfPR-1-1*, *PfPR-1-7*, *PfPR-1-9*, and *PfPR-1-10* were downregulated (Figure 5E). For qRT-PCR analysis, five representative *PfPR-1* genes (*PfPR-1-1*, *PfPR-1-3*, *PfPR-1-7*, *PfPR-1-12*, and *PfPR-1-14*) were selected, and their expression patterns were visualized. Under salt stress, *PfPR-1-1* and *PfPR-1-3* showed increasing expression trends, with *PfPR-1-1* reaching its highest expression level at 12 h and *PfPR-1-3* showing a significant increase at 12 h and remaining at a relatively high level at 24 h. *PfPR-1-7* and *PfPR-1-12* were induced at 6 h, with both showing significantly higher expression levels than those at 0 h. In contrast, *PfPR-1-14* showed a decreasing expression trend and was significantly downregulated after salt treatment (Figure 5D). Under drought stress, *PfPR-1-1* showed a decreasing expression trend, although the differences among time points were not statistically significant, whereas *PfPR-1-3* exhibited an increasing expression trend and was significantly upregulated at 12 h. *PfPR-1-7* was significantly downregulated after drought treatment, whereas *PfPR-1-12* was significantly upregulated at 12 and 24 h. *PfPR-1-14* showed a transient increase at 6 h, followed by significant downregulation at 12 and 24 h (Figure 5F). These results indicate that different *PfPR-1* genes exhibit distinct expression response patterns under salt and drought stresses in *Paulownia fortunei*.

### 2.7. Upstream Transcription Factors That Regulate PfPR-1-3 Gene

The above gene expression analysis suggested that the *PfPR-1-3* gene was significantly upregulated under both biotic and abiotic stresses, indicating its potential involvement in biotic and abiotic stress responses. Amino acid sequence homology analysis revealed that *PfPR-1-3* shared the highest sequence similarity with *Arabidopsis thaliana* AtPR-1-1 among all members of the *PfPR-1* family; therefore, *PfPR-1-3* was selected for subsequent analyses.

**Figure 6:**
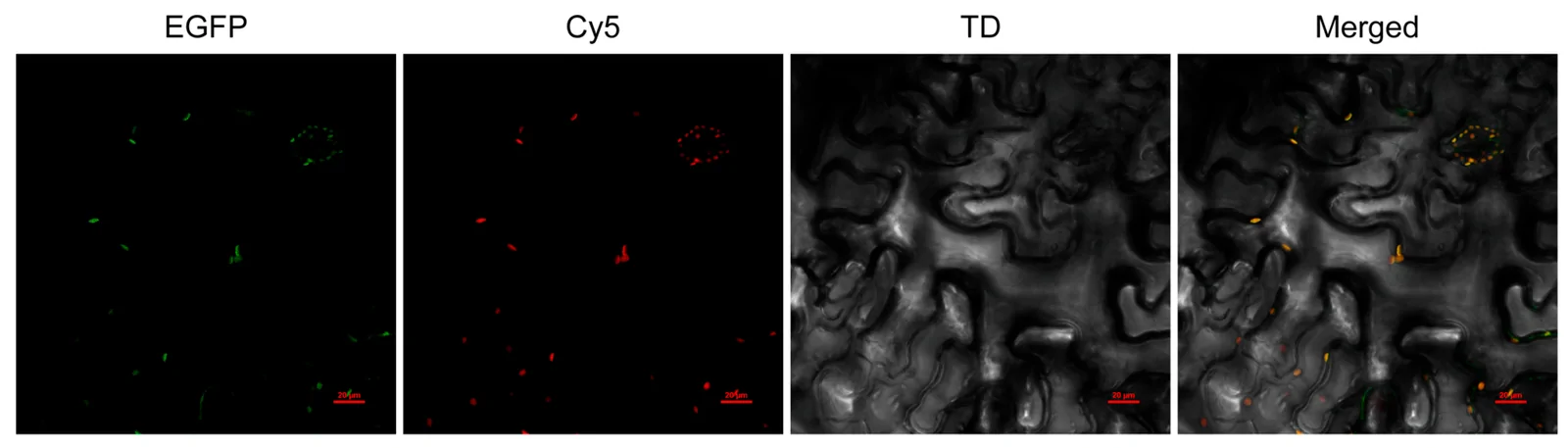
Scale bar = 20 μm, EGFP represents the green fluorescence signal of the *PfPR-1-3*–eGFP fusion protein; Cy5 represents the chloroplast autofluorescence signal; TD represents the transmitted light image showing cell morphology; and merged represents the merged images of EGFP, Cy5, and TD channels. Co-localization of EGFP fluorescence with Cy5 autofluorescence indicates the chloroplast localization of the *PfPR-1-3* protein.

To analyze the subcellular localization characteristics of *PfPR-1-3*, this study constructed a pRI101-*PfPR-1-3*-eGFP fusion expression vector (Figure 6). Expression analysis was conducted in the epidermal cells of *Nicotiana benthamiana* leaves using the transient transformation system mediated by Agrobacterium GV3101-pSoup (p19). Nuclear markers were used as controls for localization observation. Confocal microscopy observations showed that the green fluorescence signal of the *PfPR-1-3*-eGFP fusion protein completely overlapped with the red fluorescence signal emitted spontaneously by chloroplasts, indicating that *PfPR-1-3*-eGFP is localized in the chloroplast region. Unlike the commonly reported extracellular or vacuolar localization of many PR-1 proteins, the chloroplast localization observed for *PfPR-1-3* suggests that this member may be associated with chloroplast-related processes during stress responses. Chloroplasts are important sites for photosynthesis and also participate in the regulation of plant defense and stress responses through the production of reactive oxygen species and other signaling molecules. Therefore, the chloroplast localization of *PfPR-1-3* may provide a potential link between PR-1-mediated responses and chloroplast-associated defense or stress signaling. However, the biological significance of this localization remains to be experimentally determined, and further studies are needed to clarify whether *PfPR-1-3* directly participates in chloroplast-associated defense or stress-response processes.

**Figure 7:**
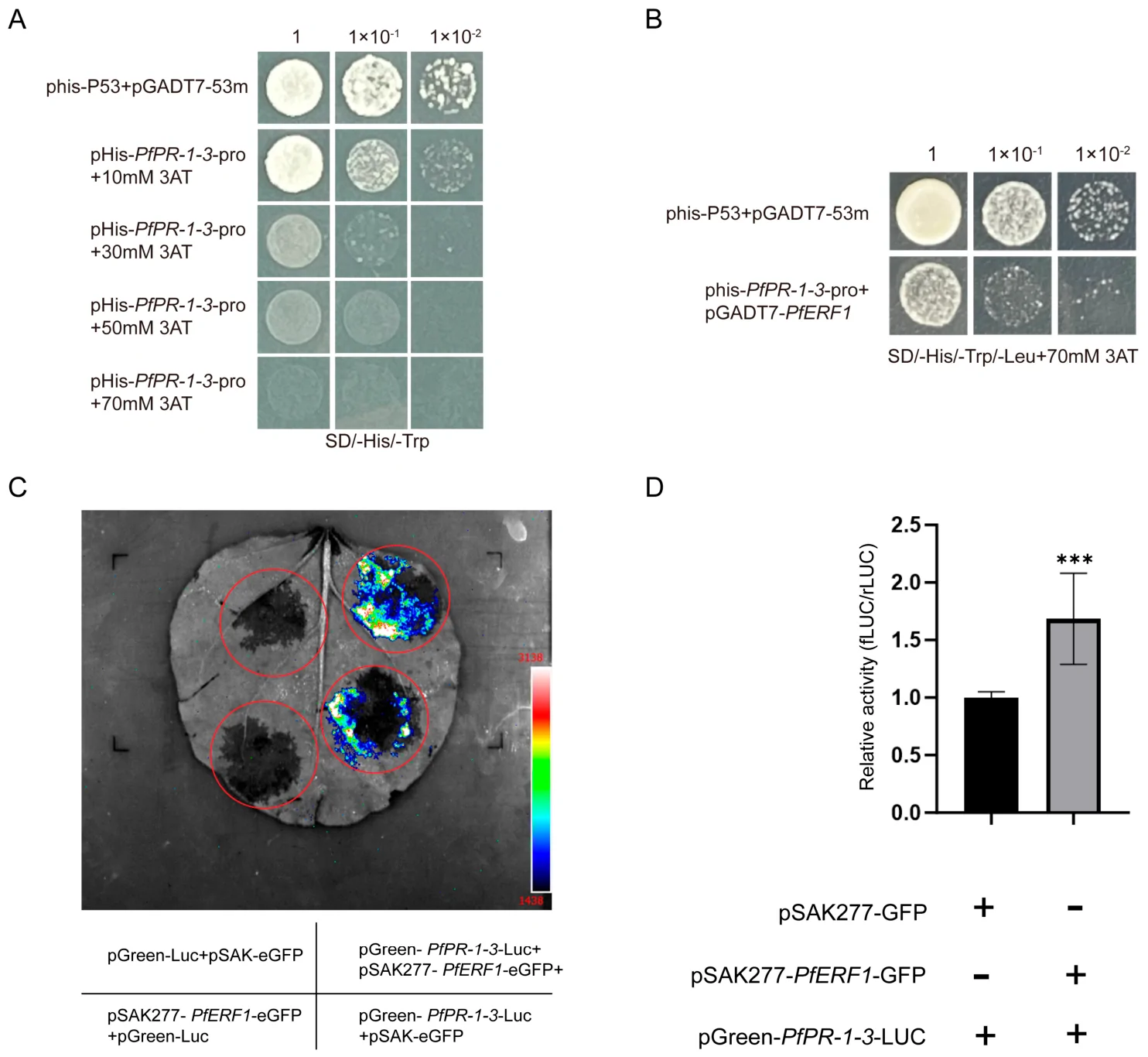
Upstream transcription factor screening and verification of the *PfPR-1-3* gene. (**A**) Autoactivation analysis of *PfPR-1-3*. (**B**) One-to-one validation of *PfERF1* binding to the *PfPR-1-3* promoter. (**C**) Observation of co-infiltrated tobacco leaves using a fully automated chemiluminescence imaging system. (**D**) Luciferase activity analysis of co-infiltrated tobacco leaves. Asterisks indicate statistically significant differences determined by Student’s *t*-test (*** *p* < 0.001).

To further identify the upstream transcription factors that regulate the expression of the *PfPR-1-3* gene, a yeast one-hybrid bait vector containing the promoter region of *PfPR-1-3*, i.e., pHis2-*PfPR-1-3*-promoter, was first constructed and subjected to autoactivation analysis. The results demonstrated that the bait vector exhibited self-activation activity, which could be effectively suppressed by the addition of 70 mM 3-AT (Figure 7A). Subsequently, upstream regulatory factors of *PfPR-1-3* were preliminarily screened through yeast one-hybrid library screening, and further point-to-point interaction verification confirmed that the transcription factor PfERF1 could bind to the promoter region of *PfPR-1-3* (Figure 7B).

To further investigate the transcriptional regulatory effect of PfERF1 on *PfPR-1-3*, a dual-luciferase reporter assay was performed using a transient tobacco expression system. The results showed that *PfERF1* significantly enhanced the transcriptional activity of the *PfPR-1-3* promoter compared with the control groups (co-transformation of the pGreen-Luc empty vector with the pSAK-eGFP empty vector, pSAK277-*PfERF1*-eGFP with the pGreen-Luc empty vector, and pGreen-*PfPR-1-3*-Luc with the pSAK-eGFP empty vector were used as controls) (Figure 7C,D). Collectively, these findings demonstrated that PfERF1 could bind to the promoter region of *PfPR-1-3* and activate its expression.

## 3. Discussion

PR-1 is one of the earliest discovered and most extensively studied members of the pathogenesis-related (PR) protein family [1]. Recent studies have demonstrated that PR-1 proteins are involved not only in disease resistance responses following pathogen infection but also in responses to abiotic stresses such as salinity, drought, and low temperature, etc. [13,27,28]. In *Paulownia fortunei*, phytoplasma infection can induce the expression of numerous defense-related genes and activate plant hormone signaling pathways [29]. However, compared with annual crops and several other woody plants, the genome-wide characteristics and stress-responsive regulatory mechanisms of the PR-1 family in *Paulownia fortunei* remain largely unexplored. In the present study, 14 members of the *PfPR-1* gene family were systematically identified in *Paulownia fortunei* for the first time. Combined analyses of phylogeny, gene structure, conserved motifs, *cis*-acting elements, and expression patterns revealed the potential involvement of the *PfPR-1* family in stress-responsive processes associated with disease resistance and salt/drought stress responses in *Paulownia fortunei*. These results provide a systematic view of the PR-1 family in *Paulownia fortunei* and extend current knowledge of PR-1-mediated stress responses in woody plants.

In this study, we found that PfPR-1 proteins generally exhibited typical characteristics of the PR-1 family, including relatively small molecular weight, high stability, and alkaline properties, and all contained a highly conserved CAP domain. Previous studies have shown that the CAP domain serves as an important functional basis for PR-1 and has been proposed to contribute to pathogen defense through mechanisms such as sterol binding [30]. However, differences were still observed among family members, suggesting that the *PfPR-1* family not only retains conserved structural characteristics but may also have undergone a certain degree of structural diversification that could contribute to functional differentiation. Most *PfPR-1* members contained relatively fewer introns, and this is a structural feature commonly observed in stress-responsive genes. Phylogenetic analysis showed that PfPR-1 proteins shared high homology with PR-1 proteins from other species, indicating strong evolutionary conservation of the PR-1 family. Similar findings have also been reported in *Arabidopsis thaliana*, *Oryza sativa*, and *Solanum lycopersicum* [31,32,33,34].

Previous studies in woody plants have mainly focused on individual PR-1 genes or their responses to specific pathogens or environmental stresses. For example, studies in *Populus deltoides* have identified *PdePR-1-10* as a pathogen-responsive PR-1 gene involved in rust resistance, whereas investigations in *Vitis vinifera* and *Eucalyptus grandis* have mainly emphasized the roles of PR-1 genes in fungal pathogen responses and defense-related processes [9,10,11]. Compared with these studies, which primarily focused on the functional characterization of individual PR-1 members under specific stress conditions, the present study provides a genome-wide characterization of the PR-1 family in *P. fortunei* and reveals the diverse expression patterns of multiple PR-1 members in response to phytoplasma infection, salt stress, and drought stress. Furthermore, this study integrates family-level analysis with upstream transcriptional regulation of a representative PR-1 gene, providing additional insights into PR-1-mediated stress responses in woody plants.

*Cis*-acting element analysis revealed that the promoter regions of *PfPR-1* genes were enriched with ABRE, TGACG-motif, CGTCA-motif, and MBS elements associated with ABA, MeJA, and stress responses, suggesting that *PfPR-1* genes may be responsive to multiple hormone- and stress-related regulatory signals. Previous studies have demonstrated that *PR-1* genes are commonly associated with SA and JA/ET signaling pathways. For example, *AtPR1* of *Arabidopsis thaliana* is an important marker gene of the SA-dependent immune pathway [35]; *CaPR1* in *Capsicum annuum* is significantly induced following *Phytophthora capsici* infection [36]; *PR-1* genes in *Nicotiana tabacum* respond both to pathogen infection and exogenous SA treatment [37]; and *OsPR1a* in *Oryza sativa* participates in plant defense responses against pathogens [38]. In addition, PR-1-related genes in *Vitis vinifera* and *Musa acuminata* have also been demonstrated to participate in pathogen defense and stress adaptation [39,40]. These studies indicate that PR-1 genes are closely associated with hormone-mediated defense and stress responses across different plant species, although the specific regulatory mechanisms may differ among species.

Expression pattern analysis showed that several *PfPR-1* members were significantly induced under salt and drought stresses, indicating that these genes may participate in stress-responsive processes in *Paulownia fortunei*. Under salt- and drought-stress conditions, plants generally produce excessive ROS, resulting in membrane lipid peroxidation and cellular damage, whereas activation of defense-related genes contributes to enhanced stress tolerance [41,42]. Our previous studies have shown that multiple stress- and defense-related genes associated with ROS accumulation, osmotic balance, and hormone signaling are differentially expressed in *Paulownia fortunei* under salt- and drought-stress conditions [43,44]. The stress-responsive expression patterns of *PfPR-1* genes observed in the present study are consistent with these previous findings and further suggest that PR-1 proteins may be involved in the broader defense and stress-response network of *Paulownia fortunei*. Similar stress-responsive expression patterns of PR-1 genes have also been reported in several plant species. For example, PR-1 family members in *Malus domestica* were reported to respond to both pathogen infection and salt stress, while PR-1 genes in other woody plants have also shown diverse expression patterns under environmental stresses [13,27,28]. Consistent with these observations, the differential expression patterns of *PfPR-1* members under salt and drought stresses suggest that PR-1-mediated stress responses may be conserved among woody plants, although individual PR-1 genes may exhibit species-specific regulatory patterns. However, the distinct expression patterns observed among individual *PfPR-1* members under salt and drought stresses suggest that their functions may not be completely redundant. Such differential responses may indicate possible functional divergence among PR-1 members, although the specific biological functions of individual genes require further validation. Therefore, although PR-1-mediated stress responses may share conserved features across plant species, the specific contribution of individual PR-1 members may differ according to species, tissue, and stress conditions.

*PR-1* genes are involved not only in abiotic stress responses but also in plant immunity. Previous studies have shown that pathogen infection can significantly induce the expression of *PR-1* genes and enhance plant disease resistance [31]. Phytoplasma infection often disrupts host hormone balance and activates immune responses. PaWB disease, caused by phytoplasma infection, severely affects the normal growth and development of *Paulownia*. Our previous studies demonstrated that phytoplasma infection significantly altered the expression of defense-related genes, TFs, and hormone signaling pathways in *Paulownia* [26]. In the present study, several *PfPR-1* members showed significant expression changes under phytoplasma infection, suggesting that *PfPR-1* genes may be associated with the response of *Paulownia fortunei* to PaWB infection. This expression response is consistent with the inducible characteristics of PR-1 genes reported in other plant–pathogen interactions. However, whether these transcriptional changes directly contribute to phytoplasma resistance or disease development remains unclear and requires further functional validation.

As important members of the APETALA2/Ethylene-Responsive Factor (AP2/ERF) family, ERF transcription factors play important roles in plant responses to both biotic and abiotic stresses. In *Arabidopsis thaliana*, ERF1 has been reported to integrate ethylene and jasmonic acid (JA) signaling and activate downstream defense-related genes, thereby enhancing resistance to *Botrytis cinerea* [45]. In *Solanum lycopersicum*, SlERF1 participates in disease defense and salt-stress responses [46], while ERF transcription factors in *Populus trichocarpa* have also been associated with responses to environmental stresses [47]. These studies suggest that ERF transcription factors may serve as important regulatory nodes connecting hormone signaling and stress-responsive gene expression. In the present study, yeast one-hybrid and dual-luciferase assays demonstrated that PfERF1 could directly bind to the promoter of *PfPR-1-3* and activate its transcription, suggesting that PfERF1 acts as an upstream transcriptional regulator of *PfPR-1-3* in *Paulownia fortunei*. Notably, the promoter of *PfPR-1-3* contained several hormone- and stress-responsive *cis*-acting elements, including elements associated with ABA and JA responses, while *PfPR-1-3* was significantly induced under both biotic and abiotic stress conditions. These findings suggest that hormone-related signals may potentially participate in the regulation of *PfPR-1-3*; however, this possibility remains a hypothesis that requires further experimental verification. The present study did not directly examine the effects of exogenous hormone treatments, hormone content changes, hormone-related mutants, or inhibitor treatments on PfERF1 and *PfPR-1-3* expression. Therefore, whether ABA, JA, ethylene, or other hormone signaling pathways are involved in PfERF1-mediated regulation remains unclear. Future studies integrating hormone treatments, biochemical analyses, and genetic approaches will be necessary to clarify the underlying regulatory mechanisms. Importantly, the current results demonstrate direct promoter binding and transcriptional activation by PfERF1, but do not establish a specific hormone-mediated signaling pathway.

Subcellular localization analysis further showed that *PfPR-1-3* was localized in the chloroplast region. The chloroplast localization of *PfPR-1-3* is noteworthy because many classical PR-1 proteins are generally associated with the secretory pathway and extracellular defense responses [27]. Therefore, the localization pattern observed for *PfPR-1-3* may indicate a different intracellular distribution from some classical PR-1 proteins. Chloroplasts are important sites for redox regulation and stress signaling in plant cells [48,49]. Based on these biological functions of chloroplasts, the chloroplast localization of *PfPR-1-3* may suggest a potential association with chloroplast-related stress responses. However, the current localization analysis only provides evidence for its subcellular distribution and does not demonstrate a direct functional role of *PfPR-1-3* in chloroplast-mediated processes. Further functional studies will be required to determine whether the chloroplast localization of *PfPR-1-3* contributes to its roles in biotic or abiotic stress responses.

In summary, this study systematically characterized the physicochemical properties, structural features, and evolutionary relationships of the *PfPR-1* gene family and analyzed the expression patterns of *PfPR-1* genes under biotic and abiotic stresses. The results indicated that multiple *PfPR-1* genes were responsive to salt stress, drought stress, and PaWB disease, suggesting their potential involvement in the stress-response and defense networks. However, the expression changes observed in this study do not directly demonstrate that these genes confer enhanced resistance or stress tolerance, and further functional validation is required. Furthermore, yeast one-hybrid and dual-luciferase assays demonstrated that PfERF1 could directly bind to and activate the *PfPR-1-3* promoter, providing evidence for a PfERF1–*PfPR-1-3* transcriptional regulatory relationship. Although the promoter of *PfPR-1-3* contains hormone-responsive elements, the present study did not experimentally verify the involvement of ABA, JA, ethylene, or other hormone signaling pathways in this regulatory relationship. In addition, *PfPR-1-3* was shown to localize to the chloroplast region, but the biological significance of this localization remains unclear. Therefore, future studies involving genetic manipulation, biochemical assays, and hormone-related experiments will be necessary to determine the specific functions of *PfPR-1-3* and further elucidate the regulatory mechanisms underlying PfERF1-mediated regulation. Overall, this study provides a systematic framework for understanding the PR-1 gene family in *Paulownia fortunei* and offers potential candidate genes for future investigation of stress-responsive mechanisms in woody plants.

## 4. Materials and Methods

### 4.1. Identification of PfPR-1 Gene Family in Paulownia

The genome sequence and annotation files of *Paulownia fortunei* were obtained from our laboratory genome database. The protein sequences of the *Arabidopsis thaliana* PR-1 family were downloaded from The Arabidopsis Information Resource (TAIR, accessed on 8 October 2024, https://www.arabidopsis.org/) based on the TAIR10/Araport11 reference annotation and used as query sequences for local BLASTP searches against the *P. fortunei* protein database using BLAST+ v2.12.0, with an E-value threshold of 1 × 10^−5^. The candidate proteins identified by BLASTP were subsequently subjected to reciprocal BLASTP searches against the reference PR-1 protein sequences to further verify their sequence similarity to known PR-1 proteins. Meanwhile, the Hidden Markov Model (HMM) profile of the CAP domain (PF00188.30) was downloaded from the Pfam database (Accessed on 8 October 2024, https://pfam.xfam.org/) and used to identify candidate PfPR-1 proteins using HMMER v3.3 with an E-value cutoff of 1 × 10^−5^. The results obtained from BLASTP and HMMER searches were integrated, and redundant sequences were removed. Candidates supported by either sequence similarity or HMM-based domain detection were retained for subsequent manual curation and domain verification.

To ensure the accuracy and reliability of *PfPR-1* gene family identification, all candidate protein sequences were manually curated. The completeness of the predicted open reading frames (ORFs), the presence of start and stop codons, and the integrity of the PR-1/CAP conserved domain were carefully examined. Redundant sequences, alternative isoforms, and incomplete protein sequences lacking a complete CAP domain were excluded. The remaining candidate proteins were further verified using SMART (Accessed on 11 October 2024, http://smart.embl-heidelberg.de/), the NCBI Conserved Domain Database (CDD, accessed on 11 October 2024, https://www.ncbi.nlm.nih.gov/cdd/), and InterProScan (Accessed on 11 October 2024, https://www.ebi.ac.uk/interpro/) to confirm the presence and integrity of the complete CAP (Cysteine-rich secretory proteins, Antigen 5, and Pathogenesis-related 1) conserved domain. For candidates showing ambiguous characteristics, particularly those with sequence similarity to the broader CAP superfamily but unclear assignment to the PR-1 family, family membership was determined based on the combined evidence from BLASTP and reciprocal BLASTP similarity, HMM-based domain detection, conserved-domain annotation, and phylogenetic placement. Candidates lacking convincing PR-1-related sequence similarity or showing incomplete or inconsistent conserved-domain characteristics were excluded. Thus, the final PfPR-1 set included only candidates with consistent sequence-similarity, conserved-domain, and phylogenetic evidence supporting their assignment to the PR-1 family. Only proteins containing an intact CAP domain and meeting the above sequence and phylogenetic criteria were retained for subsequent analyses.

The physicochemical properties of the identified PfPR-1 proteins, including amino acid length, molecular weight (MW), theoretical isoelectric point (pI), instability index, aliphatic index, and grand average of hydropathicity (GRAVY), were predicted using ExPASy ProtParam (Accessed on 14 October 2024, https://web.expasy.org/protparam/) with default parameters. Subcellular localization was predicted using WoLF PSORT (Accessed on 14 October 2024, https://wolfpsort.hgc.jp/) under default settings. Nuclear localization signals (NLSs) were identified using cNLS Mapper with default parameters, and transmembrane helices were predicted using TMHMM v2.0 (Accessed on 14 October 2024, http://www.cbs.dtu.dk/services/TMHMM-2.0/) with default parameters.

### 4.2. Phylogenetic Analysis of PfPR-1 Proteins

The PR-1 protein sequences from *Paulownia fortunei*, *Arabidopsis thaliana*, *Populus trichocarpa*, *Sesamum indicum*, and *Oryza sativa* were downloaded from the National Center for Biotechnology Information (NCBI) database (Accessed on 4 October 2026, https://www.ncbi.nlm.nih.gov/) and used for phylogenetic analysis, aligned using ClustalW implemented in MEGA7 with default parameters. A phylogenetic tree was constructed using the neighbor-joining (NJ) method based on the p-distance model with Poisson correction. The reliability of the tree topology was evaluated using 1000 bootstrap replicates, and the corresponding bootstrap values were displayed at the branch nodes. Gaps and ambiguous positions were treated using the pairwise deletion option. To further evaluate the robustness of the phylogenetic relationships, a maximum likelihood (ML) tree was additionally constructed using the same aligned protein sequences with 1000 bootstrap replicates. The major clade relationships obtained from the NJ and ML analyses were compared, and consistent topological patterns were used to support the classification of PR-1 proteins into major evolutionary clades. The phylogenetic tree was visualized and further edited using Evolview v2 (Accessed on 4 October 2026, https://www.evolgenius.info/evolview-v2). All phylogenetic analyses were performed using MEGA7.

### 4.3. Conserved Domain Analysis of PfPR-1 Members

Conserved motifs of *PfPR-1* proteins were identified using the MEME Suite (Accessed on 11 October 2026, https://meme-suite.org/meme). The maximum number of motifs was set to 10, with motif widths ranging from 6 to 50 amino acids, and the motif distribution mode was set to Zero or One Occurrence Per Sequence (ZOOPS); all other parameters were kept at their default settings. A total of 10 conserved motifs were identified. Conserved domain information of *PfPR-1* proteins was retrieved from the NCBI Conserved Domain Database (CDD) (Accessed on 11 October 2026, https://www.ncbi.nlm.nih.gov/Structure/bwrpsb/bwrpsb.cgi), and TBtools software was used to visualize the distribution of conserved motifs and domains in *PfPR-1* proteins [50].

### 4.4. Cis-Acting Element Analysis of PfPR-1 Promoters

The promoter sequences of *PfPR-1* genes were extracted from the *P. fortunei* genome, consisting of the 2000 bp upstream region of the translation initiation site (ATG). These promoter sequences were submitted to the PlantCARE database (Accessed on 11 October 2026, http://bioinformatics.psb.ugent.be/webtools/plantcare/html/) to identify potential cis-acting regulatory elements. The identified elements were classified into different categories, including hormone-responsive elements, stress-responsive elements, light-responsive elements, and growth/development-related elements. The distribution of cis-acting elements was visualized using TBtools.

### 4.5. Chromosomal Localization and Collinearity Analysis of PfPR-1

The chromosomal location information of the identified *PfPR-1* genes was obtained from the corresponding GFF3 annotation files of *Paulownia fortunei*. The chromosomal distribution map of *PfPR-1* genes was generated using TBtools. Collinearity analysis was performed using TBtools based on the MCScanX algorithm. The homologous gene pairs were identified using BLASTP results with an E-value threshold of 1 × 10^−5^, and syntenic relationships among *PfPR-1* genes were visualized using TBtools with default parameters.

### 4.6. Expression Pattern Analysis of PfPR-1 Genes Under Biotic and Abiotic Stress

Based on previously obtained transcriptome sequencing data deposited in the NCBI Sequence Read Archive (SRA) database (accession numbers: SRR11787922–SRR11787928, SRR11787942–SRR11787944, and SRR11787883), the expression patterns of *PfPR-1* genes under drought stress, salt stress, and phytoplasma infection were analyzed. These transcriptome datasets were generated previously by our laboratory for genome-wide transcriptomic profiling of *Paulownia fortunei* under different stress conditions, rather than specifically for the *PfPR-1* gene family. The drought- and salt-stress datasets were derived from previously established drought- and salt-stress experiments of *P. fortunei*, whereas the phytoplasma-related datasets were obtained by comparing healthy and naturally phytoplasma-infected plants. Leaf tissues were used for transcriptome analysis.

For the drought-stress experiment, *P. fortunei* seedlings were subjected to water-deficit treatment, with soil relative water content maintained at approximately 25% for the drought treatment and approximately 75% for the control condition. Leaf samples were collected after the designated drought-treatment period according to the original experimental design. For the salt-stress experiment, plants were treated with 200 mmol·L^−1^NaCl, with untreated plants used as controls. Three biological replicates were included for each salt-treatment condition. For the phytoplasma-related transcriptome experiment, healthy and naturally phytoplasma-infected *P. fortunei* seedlings were analyzed, with three biological replicates for each condition. For sample processing, leaf tissues were collected at the designated sampling points, immediately frozen in liquid nitrogen, and stored at −80 °C until RNA extraction. Total RNA was extracted from the collected samples, and RNA quality and quantity were evaluated before transcriptome library construction and high-throughput sequencing. The resulting sequencing data were deposited in the NCBI SRA database. For RNA-seq data processing, raw sequencing reads were subjected to quality control to remove adaptor sequences, low-quality reads, and other low-quality sequences, generating clean reads. The clean reads were aligned to the *P. fortunei* reference genome using BWA, and transcript abundance was quantified using the RSEM v1.3.0 (RNA-Seq by Expectation Maximization) software package. Gene expression levels were represented as FPKM (Fragments Per Kilobase of transcript per Million mapped reads) values.

Differentially expressed genes were identified according to the criteria of |log2 (Fold Change)| ≥ 1 and false discovery rate (FDR) < 0.05. For the present study, the FPKM values of the identified *PfPR-1* genes were extracted from the genome-wide transcriptome datasets for comparative expression analysis. The expression values were transformed using log2 (FPKM + 1), followed by hierarchical clustering. Heatmaps were generated using TBtools to visualize the expression profiles of *PfPR-1* genes under drought stress, salt stress, and phytoplasma infection. The RNA-seq datasets were generated in previous independent experiments and were reanalyzed in the present study; therefore, the stress-treatment conditions and sampling schemes of the RNA-seq datasets were not identical to those used in the independent qRT-PCR experiments.

### 4.7. Real-Time Quantitative PCR (qRT-PCR)

The plant materials used in this study included 30-day-old tissue culture-derived rooted seedlings of *Paulownia fortunei* and rooted diseased seedlings with axillary bud proliferation, which were maintained at the Paulownia Research Institute of Henan Agricultural University. Healthy *P. fortunei* seedlings and witches’ broom-diseased seedlings were directly collected from the culture materials for subsequent analysis. The diseased seedlings exhibited typical symptoms of Paulownia witches’ broom disease, including excessive axillary bud proliferation, clustered shoots, and abnormal growth phenotypes.

For abiotic stress treatments, healthy *P. fortunei* tissue culture seedlings were first acclimatized for 7 days and then transplanted into pots. After 30 days of growth, seedlings with uniform growth status were selected for stress treatments. The seedlings were grown in plastic pots (7 × 7 × 10 cm) containing humus soil mixed with vermiculite for salt stress treatment. For drought stress treatment, seedlings were transferred to a hydroponic culture containing 1/2 Murashige and Skoog (MS) nutrient solution supplemented with PEG 4000. For each abiotic stress treatment, 60 uniformly growing seedlings were selected, and seedlings with similar growth status were selected, numbered, and randomly assigned to different treatment groups before stress application to minimize variation caused by initial plant growth differences. Plants were grown under controlled conditions with a temperature of 25 ± 2 °C, relative humidity of 60–70%, photosynthetic photon flux density (PPFD) of 130 μmol·m^−2^·s^−1^, and a 16 h light/8 h dark photoperiod.

For salt-stress treatment, seedlings were irrigated with 5% (*w/v*) NaCl solution. Each pot received 100 mL of NaCl solution during salt-stress treatment. For drought stress treatment, seedlings were subjected to hydroponic culture containing 15% (*w/v*) polyethylene glycol 4000 (PEG 4000) solution to simulate drought conditions. Stress treatments were continuously applied for 24 h, and for each treatment, samples were collected at 0, 6, 12, and 24 h after treatment. At each sampling time point, root, stem, leaf, and apical bud tissues were collected from individual plants, immediately frozen in liquid nitrogen, and stored at −80 °C until RNA extraction. Three independent biological replicates were used, with each biological replicate corresponding to one individual plant. For each individual plant, root, stem, leaf, and apical bud tissues were collected separately rather than directly pooled. Total RNA was independently extracted from each tissue, and the RNA concentration of each sample was determined. The RNA samples were then normalized to the same concentration, and equal volumes of RNA from the root, stem, leaf, and apical bud of the same plant were pooled to generate one mixed-tissue RNA sample. Thus, each mixed-tissue RNA sample represented one individual plant, and the three mixed-tissue RNA samples constituted three independent biological replicates.

The rationale for pooling these tissues was to obtain an integrated expression profile representing the overall response of the PfPR-1 gene family at the whole-plant level under different stress conditions, rather than to characterize tissue-specific expression patterns. Root, stem, leaf, and apical bud tissues were included because they represent major vegetative tissues of the plant and may collectively contribute to the plant’s response to pathogen infection and abiotic stresses. Standardizing the RNA concentrations before pooling was intended to ensure a relatively balanced contribution of the four tissues to the final mixed-tissue RNA sample. We acknowledge that this approach may mask tissue-specific differences in PfPR-1 expression and therefore was not intended for the analysis of tissue-specific expression patterns.

The witches’ broom-diseased *P. fortunei* seedlings used in this study were naturally infected materials maintained in the Paulownia Research Institute of Henan Agricultural University. Disease diagnosis was performed based on typical witches’ broom symptoms and previous reports describing Paulownia witches’ broom disease caused by phytoplasma infection. Healthy seedlings without visible disease symptoms were used as controls.

To validate the transcriptome results, qRT-PCR analysis was performed on selected differentially expressed *PfPR-1* genes. Gene-specific primers for *PfPR-1* genes and the internal reference gene *Actin* were designed using Primer Premier 6.25 (Table 2). Total RNA was extracted from *Paulownia fortunei* seedlings using the CTAB method and treated with DNase I to remove genomic DNA contamination. The quality and concentration of RNA were assessed using a NanoDrop spectrophotometer and agarose gel electrophoresis. First-strand cDNA was synthesized from 1 μg of total RNA using the PrimeScript RT Reagent Kit (Takara Bio Inc., Kusatsu, Shiga, Japan) according to the manufacturer’s instructions. qRT-PCR was performed on an Applied Biosystems™ 7500 Fast Real-Time PCR System (Thermo Fisher Scientific, Waltham, MA, USA) using 2× RealStar Universal SYBR qPCR Mix by Beijing Kangrun Chenye Biotechnology Co., Ltd. (GenStar, Beijing, China). Each reaction was performed in a 20 μL reaction volume containing 10 μL of 2× SYBR qPCR Mix, 0.4 μL of each primer (10 μM), 2 μL of diluted cDNA template, and 7.2 μL of nuclease-free water. The amplification program was as follows: 95 °C for 2 min; followed by 40 cycles of 95 °C for 15 s, 60 °C for 30 s, and 72 °C for 30 s. A melting curve analysis was performed from 65 °C to 95 °C to verify the specificity of amplification.

Relative expression levels were calculated using the 2^−ΔΔC^_t_ method [51], with *Actin* used as the internal reference gene. All experiments were performed using three independent biological replicates and three technical replicates. Statistical analyses were performed using SPSS v21.0. The data were subjected to one-way analysis of variance (ANOVA), followed by Tukey’s HSD multiple comparisons test to determine significant differences among treatments. Differences were considered statistically significant at *p* < 0.05. Different lowercase letters indicate significant differences among the corresponding treatment groups.

### 4.8. Subcellular Localization

To determine the subcellular localization of *PfPR-1-3*, the coding sequence (CDS) of *PfPR-1-3* without the stop codon was amplified and fused in-frame with the eGFP reporter gene in the pRI101 vector to generate the pRI101-*PfPR-1-3*-eGFP fusion construct. The recombinant plasmid was verified by PCR and sequencing. The plasmid vector was provided by the Institute of Paulownia, Henan Agricultural University (Zhengzhou, Henan, China). The recombinant pRI101-*PfPR-1-3*-eGFP plasmid was introduced into *Agrobacterium tumefaciens* GV3101 (pSoup-p19), which was provided by Weidi Biotechnology Co., Ltd. (Shanghai, China). Positive *Agrobacterium* colonies were cultured in LB medium containing kanamycin at 28 °C for 2–3 days. The bacterial suspension was collected and adjusted to an OD600 of approximately 0.8. The *Agrobacterium* suspension containing pRI101-*PfPR-1-3*-eGFP was infiltrated into the leaves of four-week-old potted *Nicotiana benthamiana* plants. Plants transformed with the empty pRI101-eGFP vector were used as the control. At 48–72 h after infiltration, GFP fluorescence was observed using a confocal laser scanning microscope. GFP fluorescence was detected in the EGFP channel, while chloroplast autofluorescence was detected in the Cy5 channel. The fluorescence signals were merged to determine the subcellular localization of the *PfPR-1-3*-eGFP fusion protein. Images of the experimental and control groups were acquired under the same or appropriately matched imaging conditions.

### 4.9. Yeast One-Hybrid Assay

The 2000 bp promoter region upstream of the translation initiation codon (ATG) of *PfPR-1-3* was amplified from *Paulownia fortunei* genomic DNA and cloned into the pHis2 vector to generate the bait construct pHis2-*PfPR-1-3*-pro. The pHis2 and pGADT7 vectors used in this study were provided by the Institute of Paulownia, Henan Agricultural University (Zhengzhou, Henan, China). The recombinant pHis2-*PfPR-1-3*-pro vector was transformed into the yeast strain Y187 provided by the Institute of Paulownia, Henan Agricultural University (Zhengzhou, Henan, China) for autoactivation analysis. Yeast cells were cultured on YPDA medium at 30 °C for 3–5 days. The self-activation activity of the bait vector was evaluated on selective medium supplemented with different concentrations of 3-amino-1,2,4-triazole (3-AT). Based on the autoactivation assay, 70 mM 3-AT was selected for subsequent yeast one-hybrid assays. The coding sequence (CDS) of *PfERF1* was amplified from the pRI101-*PfERF1*-eGFP plasmid and inserted into the pGADT7 vector to generate the prey construct pGADT7-*PfERF1*. The bait and prey vectors were co-transformed into Y187 yeast cells, and transformants were screened on SD/-Trp/-Leu/-His-selective medium. Yeast growth was used to determine the binding activity of PfERF1 to the *PfPR-1-3* promoter.

### 4.10. Dual-Luciferase Reporter Assay

Using pHis2-*PfPR-1-3*-pro and pRI101-*PfERF1*-eGFP as templates, the promoter sequence of *PfPR-1-3* and the CDS of *PfERF1* were amplified, respectively. The *PfPR-1-3* promoter fragment was inserted into the pGreenII 0800-LUC vector to generate the reporter construct pGreenII-*PfPR-1-3*-pro-LUC, while the *PfERF1* CDS was cloned into the pSAK277 vector to generate the effector construct 35S:*PfERF1*-GFP. All plasmid vectors used in this study were provided by the Paulownia Research Laboratory. The recombinant constructs were verified by colony PCR and sequencing. The recombinant reporter and effector vectors were separately transformed into *Agrobacterium tumefaciens* GV3101 harboring pSoup-p19, which was provided by Weidi Biotechnology Co., Ltd. Positive *Agrobacterium* colonies were cultured in LB medium containing kanamycin, spectinomycin, and rifampicin at 28 °C for 2–3 days. The bacterial suspensions were collected and adjusted to an OD_600_ of approximately 0.8. The *Agrobacterium* cultures containing the reporter and effector constructs were mixed at a volume ratio of 5:1 and co-infiltrated into the leaves of four-week-old potted *Nicotiana benthamiana* plants. At 3 days after infiltration, luciferase activity was detected using a Dual-Luciferase Reporter Gene Assay Kit (11402ES60, 100 assays). Firefly luciferase (LUC) and Renilla luciferase (REN) activities were measured according to the manufacturer’s instructions, and the LUC/REN ratio was calculated to evaluate the transcriptional activation activity of PfERF1 on the *PfPR-1-3* promoter. Sodium salt luciferase substrate used for luminescence detection was purchased from Beijing Solarbio Science & Technology Co., Ltd. (Beijing, China). Luciferase signals were recorded using an automated chemiluminescence imaging system Tanon 5200 automated chemiluminescence imaging system (Tanon, Shanghai, China). Three control combinations were included: pGreenII 0800-LUC empty vector + pSAK277-GFP empty vector, pSAK277-*PfERF1*-GFP + pGreenII 0800-LUC empty vector, and pGreenII-*PfPR-1-3*-pro-LUC + pSAK277-GFP empty vector. The LUC/REN ratio of the empty-vector control was normalized to 1. The experiment was performed with three independent biological replicates.

### 4.11. Statistical Analysis

All experimental data were analyzed using SPSS Statistics v21.0 (IBM Corp., Armonk, NY, USA). The qRT-PCR experiments were performed with three independent biological replicates (*n* = 3), and each biological replicate included three technical replicates. The relative expression levels of *PfPR-1* genes were calculated using the 2 ^-ΔΔCt method. Data are presented as the mean ± standard deviation (SD).

For statistical comparisons among multiple groups, one-way analysis of variance (one-way ANOVA) followed by Tukey’s HSD multiple comparisons test was performed to determine significant differences among groups. Different lowercase letters (a, ab, b, bc, and c) indicate significant differences among treatments (*p* < 0.05), where means sharing at least one common letter are not significantly different.

For comparisons between two groups, statistical significance was evaluated using Student’s *t*-test, with *p* < 0.05 considered statistically significant.

## 5. Conclusions

In this study, the *PfPR-1* gene family in *Paulownia fortunei* was systematically identified and analyzed. The results showed that all PfPR-1 proteins contained the typical CAP conserved domain and exhibited high evolutionary conservation. Most *PfPR-1* members possessed few introns, and their promoter regions contained abundant *cis*-acting elements related to hormone signaling, abiotic stress responses, and pathogen defense, suggesting that they may be involved in responses to both biotic and abiotic stresses in *Paulownia fortunei*. Expression analysis demonstrated that most *PfPR-1* genes were differentially expressed under phytoplasma infection, salt and drought stresses, suggesting their potential involvement in stress responses. In addition, distinct expression patterns among different members revealed that individual *PfPR-1* genes may exhibit different response patterns under various stress conditions. Furthermore, yeast one-hybrid and dual-luciferase assays demonstrated that the transcription factor PfERF1 could directly bind to the promoter of *PfPR-1-3* and activate its expression, indicating that PfERF1 acts as an important upstream regulator of *PfPR-1-3* expression. Combined with promoter element analysis, it is speculated that ethylene/jasmonic acid-related signaling may be involved in the regulation of *PfPR-1-3* expression by PfERF1; however, this hypothesis requires further experimental verification. Overall, this study preliminarily revealed the structural characteristics, expression patterns, and transcriptional regulatory mechanisms of the *PfPR-1* gene family, providing a theoretical basis for further elucidating the molecular mechanisms underlying stress responses in *Paulownia fortunei*.

## Figures and Tables

**Table 1:** Information and physicochemical characteristics of PfPR-1 family in *Paulownia fortunei*.

Gene ID	Gene Name	Length (aa)	MW (kDa)	pI	Instability Index	Aliphatic Index	GRAVY	Subcellular Localization	Formula
Pfo01g008290.1	*PfPR-1-1*	168	18.80	9.03	34.79	74.46	−0.22	Extracellular	C_841_H_1264_N_240_O_234_S_10_
Pfo02g015440.1	*PfPR-1-2*	163	18.20	8.90	44.52	61.66	−0.42	Extracellular	C_800_H_1199_N_239_O_230_S_11_
Pfo02g015450.1	*PfPR-1-3*	163	18.41	6.40	28.31	68.16	−0.43	Chloroplast	C_814_H_1218_N_232_O_240_S_10_
Pfo02g015460.1	*PfPR-1-4*	163	17.99	6.86	26.17	72.94	−0.23	Extracellular	C_799_H_1189_N_219_O_242_S_8_
Pfo03g003240.1	*PfPR-1-5*	226	25.85	7.57	39.77	74.69	−0.35	Mitochondria	C_1161_H_1759_N_309_O_335_S_14_
Pfo03g003250.1	*PfPR-1-6*	164	18.72	8.47	39.03	57.87	−0.46	Chloroplast	C_839_H_1229_N_237_O_233_S_11_
Pfo03g003260.1	*PfPR-1-7*	169	19.30	8.47	39.66	62.49	−0.39	Chloroplast	C_866_H_1277_N_243_O_239_S_12_
Pfo04g003290.1	*PfPR-1-8*	161	18.12	5.89	40.52	63.60	−0.39	Extracellular	C_799_H_1187_N_227_O_237_S_11_
Pfo04g003300.1	*PfPR-1-9*	157	17.68	5.48	40.15	71.40	−0.32	Cytoplasm	C_779_H_1155_N_219_O_232_S_12_
Pfo06g011890.1	*PfPR-1-10*	179	19.39	8.78	38.66	69.83	−0.34	Chloroplast	C_857_H_1333_N_239_O_257_S_9_
Pfo13g007580.1	*PfPR-1-11*	169	18.99	5.24	50.46	78.46	−0.24	Chloroplast	C_853_H_1285_N_221_O_254_S_9_
Pfo17g002180.1	*PfPR-1-12*	161	18.03	9.37	40.34	72.73	−0.30	Chloroplast	C_792_H_1202_N_244_O_223_S_10_
Pfoxxg008850.1	*PfPR-1-13*	203	23.40	8.75	42.34	72.51	−0.42	Chloroplast	C_1057_H_1599_N_293_O_290_S_11_
Pfoxxg016540.1	*PfPR-1-14*	147	17.02	8.42	38.15	61.02	−0.42	Extracellular	C_762_H_1137_N_207_O_211_S_14_

Length, amino acid sequence length; MW, molecular weight; pI, isoelectric point; and GRAVY, grand average of hydropathicity.

**Table 2:** The primers and reference genes that were used in this study.

Gene	Gene Name	Forward Primer (5′–3′)	Reverse Primer (5′–3′)	Amplicon	
				L (bp)	Tm (°C)
Pfo01g008290.1	*PfPR-1-1*	AACATTTTCTGGGGCAGCGG	GCACCCAATTCTTGTCGTTCC	168	60.0
Pfo02g015450.1	*PfPR-1-3*	GCAGCTTTTGCCCGAAACTA	ATTAGGCTTCTCCCCAACCC	163	60.0
Pfo02g015460.1	*PfPR-1-4*	TGCGCTCTAACCCACTCCTA	ACCACTTGCGTGTAGTGCAA	163	60.0
Pfo03g003240.1	*PfPR-1-5*	GTGCCTGGACAGATGAGGAG	CGAGCACACCCGATTTTTCG	226	60.0
Pfo03g003260.1	*PfPR-1-7*	GAAACTAGAGCGGTACGCGG	CCTCTCCATAGGCACACGAA	169	60.0
Pfo04g003290.1	*PfPR-1-8*	GTGCCGAAGTAGGGGTTCAA	CTTCTCCTCCACCCACAACG	161	60.0
Pfo17g002180.1	*PfPR-1-12*	TGCCCAAGCTCAAAACACAC	CGAGGTTCTCACCGTATGGC	161	60.0
Pfoxxg016540.1	*PfPR-1-14*	AGGTCATTCACTCGTTCCCG	ATCTGCGTGTAATGCCCACA	147	60.0
Pfo02g015450.1	pRI101-*PfPR-1-3*	CTTCACTGTTGATACATATGATGGAAATCCACAAGAAGTTTTCA	CTTGCTCACCATGGATCCCTAGTAAGGACGTTCACCAATGTAATT	492	82.1
Pfo02g015450.1	phis-*PfPR-1-3*	CGACTCACTATAGGGCGAATTCTCTAGAGTCAGCTAAGTACTTTTTCTTAACTC	ATTCGCGAACGCGTGAGCTCTTTGATTGGTGACAAATTGTTGG	2000	87.1
Pfo02g015450.1	pGreen-*PfPR-1-3*	GTCGACGGTATCGATAAGCTTTCTAGAGTCAGCTAAGTACTTTTTCTTAACTC	CGCTCTAGAACTAGTGGATCCTTTGATTGGTGACAAATTGTTGG	2000	81.2
Pfo13g001260.1	AD*-PfERF1*	GTACCAGATTACGCTCATATGATGGCAGCAGCTATAGATATATATGGT	ACGATTCATCTGCAGCTCGAGTTATAGAGCTGCCCAATCAATCTC	1143	83.8
Pfo13g001260.1	pSAK277*-PfERF1*	GTGGATCCAAAGAATTCATGGCAGCAGCTATAGATATATATGGT	CTCCTTTACCCATGAATTCTAGAGCTGCCCAATCAATCTCC	1143	80.5
Pfo06g005670.1	*PfActin*	AATGGAATCTGCTGGAAT	ACTGAGGACAATGTTACC	1143	60.0

L, length of PCR production (in base pairs); Tm, melting temperature (in °C).

## Data Availability

Data are contained within the article.

## Abbreviations

The following abbreviations are used in this manuscript:

PR-1	Pathogenesis-related protein 1
PaWB	Paulownia witches’ broom
ROS	Reactive oxygen species
SAR	Systemic acquired resistance
SA	Salicylic acid
JA	Jasmonic acid
MeJA	Methyl jasmonate
FPKM	Fragments Per Kilobase of transcript per Million mapped fragments
TF	Transcription factor
AP2/ERF	APETALA2/Ethylene-Responsive Factor
LUC	Firefly luciferase
REN	Renilla luciferase
